# Long-Term Recurrence of Retroperitoneal Dedifferentiated Liposarcoma After a Complete Surgical Resection: A Report of a Rare Phenomenon

**DOI:** 10.7759/cureus.17003

**Published:** 2021-08-08

**Authors:** Saarang Patel, Albert Alhatem, Jimmy S Patel, Ruonan Zhang, Ravi J Chokshi

**Affiliations:** 1 Division of Surgical Oncology, Rutgers New Jersey Medical School, Newark, USA; 2 Department of Pathology, Immunology, and Laboratory Medicine, Rutgers New Jersey Medical School, Newark, USA; 3 Department of Dermatology, Saint Louis University School of Medicine, St. Louis, USA; 4 Department of Radiation Oncology, Emory University School of Medicine, Atlanta, USA

**Keywords:** retroperitoneal, dedifferentiated liposarcoma, recurrence, radiation therapy, complete resection, molecular testing

## Abstract

The objective of this report is to present a rare case of a recurrence after 20 years of retroperitoneal dedifferentiated liposarcoma after surgical resection and to discuss the lessons learned from this rare phenomenon for patients management and understanding the behavior of these aggressive tumors.

A 75-year-old woman presented with recurrent retroperitoneal dedifferentiated liposarcoma who had undergone a surgical resection 20 years earlier and had no evidence of disease on frequent follow-ups during that period. The histopathologic examination revealed different morphologic characteristics between the initial and recurrent presentations. The fluorescence in situ hybridization showed amplification of the mouse double minute 2 homolog (MDM2), a regulator of p53 gene on chromosome 12q15, and positive cyclin-dependent kinase 4 (CDK4) immunostain.

Liposarcoma long-term recurrence is a challenging surgical disease to provide the best survival outcome. Incomplete resection could explain the recurrence in anatomic locations where the lesions are intermixed with the neighboring adipose tissue. However, dedifferentiated liposarcoma can rarely recur after 20 years. The molecular transformation and the survival analysis of these tumors predict certain behaviors. The refraction for radiation therapy in our case and the mixed morphology provide some insight into the biology and the clinical management for these aggressive tumors.

## Introduction

Liposarcomas are rare malignant tumors that are derived from mesenchymal cells, specifically adipocytes, and are classified as the most common soft tissue sarcoma making up about 20% of all types of adult sarcomas [1]. As defined by the World Health Organization (WHO), liposarcoma is divided into three groups, which include well-differentiated and dedifferentiated liposarcoma (WDL/DDL), myxoid liposarcoma (MRCL), and pleomorphic liposarcoma (PLS) [1].

The first reported case of a retroperitoneal liposarcoma in the literature was published in 1949 [2]. Pathologically, WDL shows similar features to lipoma with a mixture of both normal adipocytes intermixed with atypical adipocytes [3]. DDLs are characterized clinically as a WDL that has dedifferentiated into a sarcoma that expresses genetic changes within 6q23 and 1p32 coamplifications [3]. 

The gold standard of care for retroperitoneal liposarcoma is aggressive surgical management, however, radiation or chemotherapy are also possible treatment options [4]. In addition to the traditional treatment options, promising early clinical trial results with the use of cyclin-dependent kinase 4 (CDK4) inhibitor palbociclib have shown to help inhibit the growth of tumor cells, and have resulted in improved clinical outcomes for both WDL and DDL [5]. The five-year disease survival rate for DDLs is 44% and the location is a major indication of the disease prognosis [6]. Although at this time the role of pre-operative radiotherapy is still unclear, clinical trials are actively investigating its exact use in treating retroperitoneal liposarcoma.

From a clinical perspective, a retroperitoneal liposarcoma recurrence following the initial treatment plan is not uncommon, as disease progression within six months to two years has been frequently noted [7]. Throughout a patient’s routine CT scan follow-up during the duration of their care, the progression would not only appear at the initial site of the resection but also throughout the abdomen [7]. With recurrent retroperitoneal liposarcomas growing at a relatively fast pace, follow-up intervals for patients tend to be around three months [7].

We report a case of a 75-year-old female with a past medical history of retroperitoneal liposarcoma, however, although resection was done on this patient, a new retroperitoneal liposarcoma regrowth was found 20 years post-operatively, displaying this rare phenomenon. We discuss the challenges of managing patients with long-term recurrence of retroperitoneal liposarcomas following complete surgical resection in order to share our experience with this rare phenomenon and to explore the biology behind the recurrence to improve the understanding of these aggressive tumors.

## Case presentation

**Figure 1:**
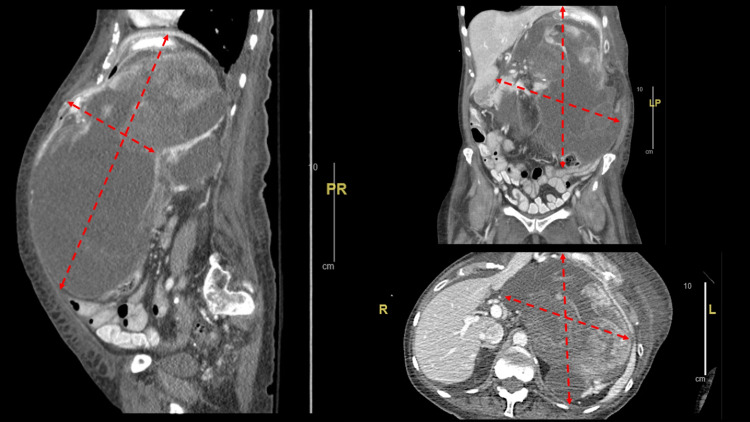
Large left retroperitoneal mass, which is predominantly cystic with some sold component and multiple septations and areas of increased attenuation at its superior portion

A 75-year-old woman with a past medical history of retroperitoneal liposarcoma and history of underlying malnutrition and hypoalbuminemia who underwent radical resection in 2000 presented in 2020 with abdominal discomfort and a CT scan showed evidence of retroperitoneal tumor mass (Figure 1). 

**Figure 2:**
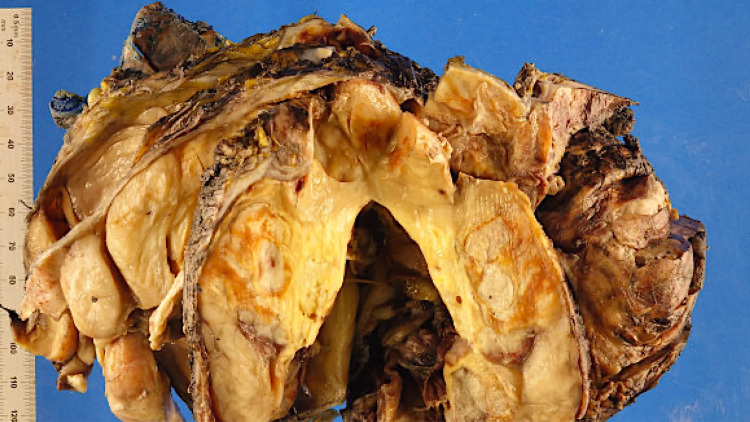
Gross image showing a multinodular mass involving the pancreas, with tan and yellow cut surfaces

There was a large left abdominal/retroperitoneal mass measuring approximately 20.5 x 22.8 x 26.1 cm (Figure 2). The mass was predominantly cystic but contained multiple septations and areas with increased attenuation. There was an associated mass effect on the adjacent abdominal organs including encasement of the pancreatic body and encasement of the distal transverse colon. There was no evidence of abnormal bowel distention to indicate obstruction. The pathology from the previous resection 20 years ago was found to be similar to the current lesion noted.

She was managed by radiation therapy (RT) at an outside institution, but cancer was refractory and was therefore admitted to our institution for surgical resection of a new left-sided abdominal mass. The patient underwent a complex operation including the following elements: exploratory laparotomy, lysis of adhesions, radical resection of abdominal mass, total gastrectomy, distal pancreatectomy and splenectomy, roux-en-Y reconstruction with neo-gastric conduit obstruction, partial colectomy, splenic flexure immobilization, appendectomy, liver wedge resection, omentectomy, and feeding jejunostomy placement. 

**Figure 3:**
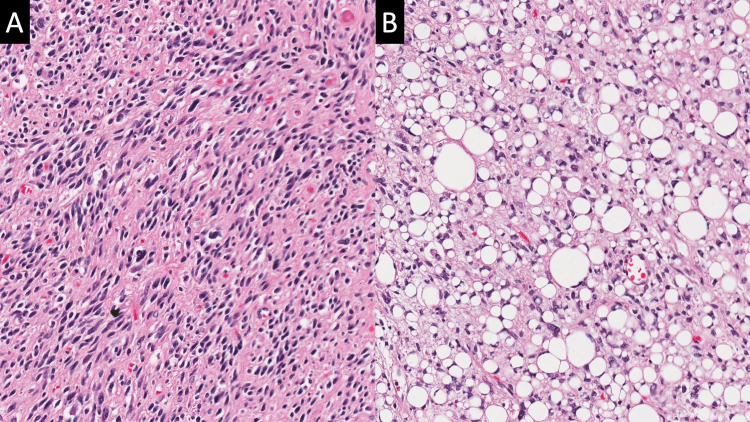
Histopathologic sections showing H&E stain of the spindle cell lesion with significant atypia and rare mitosis (A). H&E stain shows some part of the lesion as lipogenic sarcoma (B).

**Figure 4:**
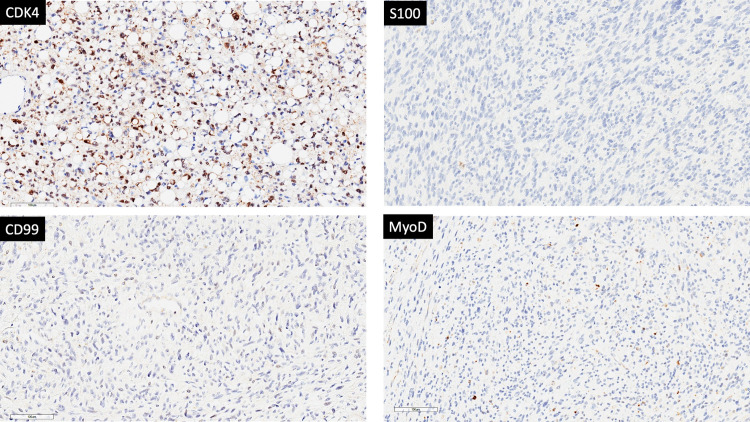
Immunohistochemistry analysis shows CDK4 positive cytoplasmic stain of the lesion cells with negative stain of CD99, S100, and MyoD. CDK4: cyclin-dependent kinase 4; MyoD: myoblast determination protein; CD99: cluster of differentiation 99

Histopathologic examination showed undifferentiated spindle cell sarcoma in the majority of the mass with dedifferentiated retroperitoneal liposarcoma components in some areas (Figure 3). Mitotic activity was low, but atypia was evident. Immunohistochemistry was noncontributory (myoblast determination protein 1 {MyoD1} occasional, spinal muscular atrophy {SMA} occasional, s100 rare, cluster of differentiation 99 {CD99} negative; Figure 4). However, fluorescence in situ hybridization showed amplification of the mouse double minute 2 homolog (MDM2) gene on chromosome 12q15 and an immunostain that was positive for CDK4, which supported the diagnosis of de-differentiated retroperitoneal liposarcoma. 

Post-operatively, the patient was transferred to the surgical intensive care unit and required vasopressors and ventilator support. Due to her underlying malnutrition and hypoalbuminemia, the patient had significant fluid shifts leading to bilateral pleural effusions which necessitated bilateral chest tubes and a tracheostomy. She was successfully weaned off these and ultimately required hemodialysis for management of acute kidney injury and metabolic encephalopathy. Once the patient was hemodynamically stable with significantly improved mental status, the patient was transferred to acute rehab, had her trach removed, and was tolerating a diet.

## Discussion

We presented a rare case of long-term recurrence of retroperitoneal dedifferentiated liposarcoma (DDL) after complete surgical resection in a 75-year-old woman. The implication of this rare phenomenon is of clinical and biological significance, as it poses a challenge from the management point of view, but it also sheds light on the biology of these aggressive tumors. Genetically WDL and DDL are similar, but DDL has more genetic alterations, specifically 6q23 and 1p32 co-amplifications [8]. Furthermore, although both WDL and DDL affect primarily middle-aged individuals, the overall disease course is different [9]. In addition, WDL can transform into DDL but does not metastasize, as oppose to DDL, which has a more aggressive clinical behavior and does metastasize [8].

The overall rate of local recurrence of DDLs is 80% within five years of a complete surgical resection [10]. In fact, it is noted that approximately 25% of all patients will form a distant metastatic disease following treatment of the primary tumor [11]. To our knowledge, the long-term recurrence of retroperitoneal liposarcomas after 20 years post complete resection has not been reported previously. In fact, a case report published by Horowitz et al. gives an inside look into the treatment method for a DDL [12]. Their particular patient was a 73-year-old male who was clinically diagnosed with a left-sided intra-abdominal mass with pathology showing a high-grade DDL with also a rhabdosarcomatous component [12]. A residual tumor of the retroperitoneal posterior margin was noted on the post-operative CT for this patient [12]. The patient’s treatment modality consisted of resection of the retroperitoneal tumor followed by both a left colectomy and left nephrectomy [12]. Although both our case report and Horowitz et al. mentioned how postoperatively a new growth was found in the patient, our particular case report outlines a new mass growth in the patient that occurred 20 years after complete resection [12]. 

Although surgical resection currently remains the optimal treatment option for patients, for those suffering from unresectable or recurrent growths, chemotherapy remains a viable option as well. In fact, the median survival rate was found to be 29 months (95% CI 24-40 months) for DDL patients following chemotherapy, demonstrating the success of chemotherapy as a treatment option [13]. Although DDL resection procedures and treatment methods have been noted in the current literature, it is important to understand how our particular case adds to the current landscape of treatment options for this rare form of cancer when new growth is found within the patient many years later. 

The treatment of choice for DDLs is the complete surgical resection of cancer which, in turn, leads to an increase in overall patient quality of life. However, patients may undergo multiple surgical operations for these tumors due to the recurrence. Parmeggiani et al. mentioned in their case report that their particular patient had to undergo three complete surgical excisions of cancer in a span of three years to increase the overall patient’s survival [13]. This proposes the question of whether achieving a complete surgical resection is indeed attainable. One explanation of the recurrence could be due to incomplete tumor resection, especially if lesions were in locations admixed with visceral adipose tissue and it is difficult to assess during the operation the real tumor extent, and therefore the true surgical margins [14].

Our patient’s tumor recurred after 20 years, which is very unlikely to be due to incomplete resection because, within a five-year period, it’s noted that 80% of all DDL recur locally [10]. This displays that although complete surgical removal was performed in our patient, a DDL growth taking place 20 years post-operatively is a rare phenomenon and displays new challenges for treating this form of sarcomas. We speculate that new genetic mutations have occurred in our patients as the new growth had both WDL and DDL, which could explain the long-term recurrence and shed new light on the biology of the retroperitoneal liposarcoma's transformation. 

## Conclusions

In summary, retroperitoneal liposarcoma long-term recurrence is a challenging aspect of the disease from a surgical point of view to provide the best outcome and to improve survival. Incomplete resection is a possibility in some anatomic locations, especially if the lesions are intermixed with neighboring adipose tissue. However, dedifferentiated liposarcoma can rarely recur after 20 years. The molecular transformation and the survival analysis of these tumors predict certain behaviors. The refraction for radiation therapy in our case and the mixed morphology provide some insight into the biology and the clinical management for these aggressive tumors. 
